# Getting a grip on problem gambling: what can neuroscience tell us?

**DOI:** 10.3389/fnbeh.2014.00141

**Published:** 2014-05-20

**Authors:** Anna E. Goudriaan, Murat Yücel, Ruth J. van Holst

**Affiliations:** ^1^Department of Psychiatry and Amsterdam Institute for Addiction Research, Academic Medical Center, University of AmsterdamAmsterdam, Netherlands; ^2^Monash Clinical and Imaging Neuroscience (MCIN) Laboratory, Monash Biomedical Imaging and School of Psychological Sciences, Monash UniversityMonash, VIC, Australia; ^3^Centre for Cognitive Neuroimaging, Donders Institute for Brain, Cognition and Behaviour, Radboud University NijmegenNijmegen, Netherlands

**Keywords:** pathological gambling, disordered gambling, reward sensitivity, impulsivity, cue reactivity, response inhibition, review, addictive behaviors

## Abstract

In problem gamblers, diminished cognitive control and increased impulsivity is present compared to healthy controls. Moreover, impulsivity has been found to be a vulnerability marker for the development of pathological gambling (PG) and problem gambling (PrG) and to be a predictor of relapse. In this review, the most recent findings on functioning of the brain circuitry relating to impulsivity and cognitive control in PG and PrG are discussed. Diminished functioning of several prefrontal areas and of the anterior cingulate cortex (ACC) indicate that cognitive-control related brain circuitry functions are diminished in PG and PrG compared to healthy controls. From the available cue reactivity studies on PG and PrG, increased responsiveness towards gambling stimuli in fronto-striatal reward circuitry and brain areas related to attentional processing is present compared to healthy controls. At this point it is unresolved whether PG is associated with hyper- or hypo-activity in the reward circuitry in response to monetary cues. More research is needed to elucidate the complex interactions for reward responsivity in different stages of gambling and across different types of reward. Conflicting findings from basic neuroscience studies are integrated in the context of recent neurobiological addiction models. Neuroscience studies on the interface between cognitive control and motivational processing are discussed in light of current addiction theories.

**Clinical implications:** We suggest that innovation in PG therapy should focus on improvement of dysfunctional cognitive control and/or motivational functions. The implementation of novel treatment methods like neuromodulation, cognitive training and pharmacological interventions as add-on therapies to standard treatment in PG and PrG, in combination with the study of their effects on brain-behavior mechanisms could prove an important clinical step forward towards personalizing and improving treatment results in PG.

## Gambling, cognitive control, and impulsivity: on gambling and the concept of self-control

Pathological gambling (PG) has a relatively stable prevalence in western countries, with estimations ranging from 1.4% (lifetime prevalence) in the USA, to 2% in Canada (Welte et al., 2002; Cox et al., 2005). Prevalence rates are comparable and relatively stable between countries and across survey instruments (Stucki and Rihs-Middel, 2007), with a cumulative rate around 3% for PG and problem gambling (PrG) together.

Diminished cognitive control over the urge to engage in addictive behaviors is a central characteristic of PG. It is central to the *phenomenology* of PG as defined in several of the diagnostic criteria of PG (e.g., unsuccessful efforts to control, cut back, or stop gambling). Defined from a neurocognitive perspective, the overarching notion of cognitive control can be defined as the ability to control one’s actions. Cognitive control can be divided in several (sub) processes such as the ability to inhibit automatic responses (referred to as response inhibition, measured by tasks like the stop signal task) and the ability to ignore irrelevant interfering information (referred to as cognitive interference measured by tasks such as the Stroop task). In terms of the verbal representation of cognitive control, the term “impulsivity” is used regularly, to indicate a tendency to act on a whim, to display behavior that is characterized by little or no forethought, reflection, or consideration of the consequences (Daruna and Barnes, 1993). Impulsivity is a multi-faceted construct that often is deconstructed into the concept of “impulsive action”, characterized by diminished motor inhibition and “impulsive choice”, represented by a propensity to favor immediate rewards over delayed, larger, or more beneficial rewards in decision-making processes (Lane et al., 2003; Reynolds, 2006; Reynolds et al., 2006; Broos et al., 2012). Impaired response inhibition is thought to predispose for impulsive behavior, and diminished cognitive control has been implicated as an endophenotypic vulnerability marker for addictive disorders in recent years.

Numerous self-report and neurocognitive studies in PG indicate increased impulsivity on measures such as the Barratt Impulsiveness Scale, or Eysenck’s and Impulsiveness Questionnaire (Eysenck et al., 1985) and diminished cognitive control as evidenced in diminished response inhibition, cognitive interference, and delay discounting tasks (for reviews see: Goudriaan et al., 2004; Verdejo-Garcia et al., 2008; van Holst et al., 2010a, b). Clinically, the diminished control over one’s own behavior could lead to a higher vulnerability to develop PrG or PG, since for instance a diminished control to inhibit responses (response inhibition) could be associated with a more fast progression into PrG due to the diminished ability to stop gambling when one’s money runs out. Similarly, a diminished cognitive interference ability could lead to a diminished ability to ignore cues for gambling in the environment. For example, experiencing high cognitive interference could lead to a higher responsivity towards gambling advertisements, which could lead to a higher likelihood of engaging in gambling, whereas diminished cognitive control could result in diminished ability to stop gambling despite high losses.

Several reviews have already been published with a focus on cognitive control or impulsivity studies in PG (van Holst et al., 2010a, b; Conversano et al., 2012; Leeman and Potenza, 2012). This review therefore focuses on more recent neurocognitive and neuroimaging studies that have been published in PG and PrG. Specifically, this review also focuses on neuroimaging studies of motivational aspects (e.g., cue reactivity), cognitive functions (e.g., impulsivity), and on neuroimaging studies addressing the interaction between cognitive and motivational processes.

Whereas a clear definition of PG is present, fulfillment of the (usually latest version of the) DSM diagnostic criteria for PG, there is no clear definition for PrG. Usually, PrG refers to a less severe form of PG, or is used when no clinical diagnosis can be determined, due to the administration of questionnaires instead of structured clinical interviews. Some studies define PrG by a score of 5 or higher on the South Oaks Gambling Screen (SOGS) or by a score of 3 or higher on a short version of the SOGS (Slutske et al., 2005). In other studies gamblers who are in treatment for problematic gambling, and fulfill up to four criteria of the PG criteria, are defined as problem gamblers (Scherrer et al., 2005), or the entire studied group is defined as “problem gamblers” when not all of the participants who are in treatment fulfill five or more of the PG criteria (e.g., de Ruiter et al., 2012). In this review therefore, PrG is used, when no information is given on DSM diagnosis of PG, but when questionnaire data indicate that PrG is present.

As concluded in Conversano et al. (2012), several studies indicate diminished cognitive control in PG as evidenced in stop-signal tasks, Go-NoGo tasks, and also in Stroop task performance. Ledgerwood et al. (2012) however assessed response inhibition with a Stroop and stop signal task, and reported no differences between pathological gamblers and controls on these tasks, but differences were present in planning tasks (Tower of London) and in cognitive flexibility (Wisconsin Card Sorting Test). As the sample included both community-recruited pathological gamblers (not in treatment) and treatment-seeking pathological gamblers, differences with other studies may be related to a less severe cognitive profile in non-treatment seeking pathological gamblers. Indeed, in another study by the same group lower impulsivity scores (Barratt impulsivity Scale), lower past-year illegal behaviors, lower depression and dysthymic disorders, and lower preoccupation with gambling were present in community-recruited pathological gamblers vs. pathological gamblers in treatment (Knezevic and Ledgerwood, 2012).

Despite the number of neuropsychological studies indicating diminished cognitive control, the number of neuroimaging studies focusing on the neural mechanisms underlying diminished cognitive control is very limited and therefore all neuroimaging studies on cognitive control are discussed here. In a study by Potenza et al. a Stroop task was administered in an fMRI study in 14 pathological gamblers and 13 healthy controls (HCs) (Potenza et al., 2003a). Diminished BOLD responsivity in the left ventromedial PFC and in the superior OFC was reported in pathological gamblers compared to HCs, despite a lack of behavioral differences. This lack of behavioral differences may have been related to the modified version of the Stroop that was used: silent naming of the colors of the letters and behavioral performance measured by self-report of the participants after performing the Stroop task. In a recent study by de Ruiter et al. (2012), diminished neural responsivity after failed inhibitions was found in the anterior cingulate cortex (ACC) in 17 problem gamblers compared to 17 HCs. Of note, reduced activity was also observed following successful inhibitions in similar regions (right dorso-medial PFC bordering on ACC) HCs. In this study—similar to the study by Potenza et al.—no behavioral differences were found for the PrG group compared to the HCs, which may be related to power issues due to the smaller sample sizes of fMRI studies in PrG and PG compared to neuropsychological studies. Both these fMRI studies on cognitive control in PG and PrG show that diminished functioning of several prefrontal areas and of the ACC indicate that cognitive-control related brain circuitry functions are diminished in PG and PrG compared to HCs. These results implicate that diminished frontal functions may contribute to the pathophysiology of PG and PrG, in which diminished control over gambling behavior is central.

Another line of studies shows that impulsivity also plays an important role as vulnerability factor for the development of PrG. Several longitudinal studies in adolescents and adults from a research group from Montreal in Canada show that level of impulsivity is a predictor of both gambling and of PrG (Vitaro et al., 1997, 1999; Wanner et al., 2009; Dussault et al., 2011). Specifically, increasing impulsivity levels were associated with higher levels of PrG (Vitaro et al., 1997). In one of the more recent studies, a positive predictive link between impulsivity at age 14 and depressive symptoms and gambling problems at age 17 was present (Dussault et al., 2011). In another study using two male community samples, behavioral disinhibition and deviant peers were related to PrG, but also to substance use and delinquency, indicating similar risk factors for vulnerability to several externalizing problem behaviors (Wanner et al., 2009). These studies focused on adolescents and the predictive role of impulsivity for PrG; very recently two large-scale longitudinal birth cohort studies, investigated the role of impulsivity in early childhood and PrG during adulthood. In one of these studies (Shenassa et al., 2012), psychologists rated impulsive and shy/depressed behaviors at age 7, and related this to life-time self-reported PrG as adults, in a follow-up. Whereas impulsive behavior at age 7 predicted PrG, shy/depressed behavior did not predict PrG in adulthood, in this US based cohort of 958 offspring from the Collaborative Perinatal Project. In a large birth cohort study from Dunedin, New Zealand, temperament was assessed at age 3, and disordered gambling was assessed in this cohort when aged 21 and 32. Remarkably, children with (behaviorally and emotionally) undercontrolled temperament when aged 3 years, were more than twice as likely to evidence disordered gambling in adulthood, compared to children who were well-adjusted at age 3. This relationship was even stronger in boys compared to girls (Slutske et al., 2012). Several other studies show that impulsivity is also a vulnerability marker for engaging in gambling (Pagani et al., 2009; Vitaro and Wanner, 2011).

In conclusion, from this line of studies, there is strong evidence that impulsivity and diminished behavioral control play an important promoting role from the engagement in gambling to the development and persistence of at-risk gambling and PrG.

Given this crucial role of cognitive control in promoting gambling and PrG, evidenced from the birth cohort studies, neurocognitive studies, more neuroimaging studies in PrG and PG should focus on cognitive control, in order to illucidate what neurophysiological mechanisms may underly diminished cognitive control in problematic gambling. Thus, studying interactions between (novel) psychological, pharmacological, or neuromodulation interventions in PG, and their effect on the neurocircuitry of cognitive control in PG, is a very relevant venue for future neuroimaging and clinical intervention studies in PG (detailed in the Discussion section).

## Right on cue? Cue-reactivity studies in problem gambling

Compared to the small number of neuroimaging studies on cognitive control or impulsivity in PG and PrG, the topic of the neural mechanisms of cue-reactivity in PG and PrG is relatively well-studied. Five neuroimaging studies on cue-reactivity in PG and PrG (Potenza et al., 2003b; Crockford et al., 2005; Goudriaan et al., 2010; Miedl et al., 2010; Wölfling et al., 2011) and several studies focusing on cue reactivity relating to subjective craving and/or peripheral physiological responses in PrGs are present (Freidenberg et al., 2002; Kushner et al., 2007; Sodano and Wulfert, 2010). For the purpose of this review, we focus on the neuroimaging findings.

Of the five neuroimaging studies in PG and PrG related to cue reactivity, the first (Potenza et al., 2003b) used a cue reactivity paradigm consisting of videos designed to evoke emotional and motivational antecedents to gambling. In these videos, actors mimicked emotional situations (e.g., happy, sad), after which the actor described driving to or walking through a casino and experiencing the feeling of gambling. In this study, timeframes in which the participants experienced craving were analyzed for 10 pathological gamblers compared to eleven HCs. In all cases, this was before actual gambling cues were present and in response to the actors’ descriptions of the emotional situation (i.e., gambling scenarios). Less activation in the cingulate gyrus, (orbito) frontal cortex (OFC), caudate, basal ganglia, and thalamic areas was present in the 10 pathological gamblers compared to the 11 HCs. In another study using gambling-related videos to elicit cue-reactivity, 10 pathological gamblers and 10 HCs were compared on brain responsivity to these gambling-related videos compared to watching nature-related videos (Crockford et al., 2005). Higher activation in dorsal prefrontal areas, inferior frontal areas, the parahippocampal areas, and occipital lobe was found in pathological gamblers compared to HCs. In a subsequent fMRI cue-reactivity study, Goudriaan et al. (2010) found elevated activity of similar regions when comparing 17 pathological gamblers vs. 17 HCs using gambling-related and gambling unrelated photos. In this last study, a positive relationship was found between subjective craving for gambling in pathological gamblers and activity of the frontal and parahippocampal regions when viewing gambling pictures vs. neutral pictures. In an EEG study by Wölfling et al. (2011), 15 pathological gamblers were compared to 15 HCs on EEG responsivity to gambling pictures compared to neutral, positive and negative emotional pictures. Compared to HCs, pathological gamblers showed significantly larger late positive potentials (LPPs) induced by gambling stimuli when compared to neutral stimuli, but displayed comparable LPPs towards negative and positive emotional pictures. In contrast, in HCs there was a larger response towards positive and negative stimuli compared to both neutral and gambling stimuli. Higher LPPs were present in the parietal, central, and frontal electrodes in PGs compared to HCs, interpreted as a higher overall psychophysiological responsivity towards gambling stimuli in pathological gamblers.

Finally, in an fMRI study comparing brain responsivity towards high-risk vs. low-risk gambling situations in 12 problem gamblers vs. 12 HCs, problem gamblers showed an increased BOLD response in thalamic, inferior frontal, and superior temporal regions during high-risk trials, whereas a signal decrease in these regions during low-risk trials was present. The opposite pattern was observed in the non-problem gamblers (Miedl et al., 2010). The authors argue that this frontal-parietal activation pattern during high-risk trials compared to low-risk trials in problem gamblers reflects a cue-induced addiction memory network, triggered by gambling-related cues. The findings of this study implicate that high-risk wagers may be attractive to problem gamblers, eliciting cue-reactivity and craving, whereas low-risk wagers, representing a high chance to win a smaller amount of money may elicit higher reward expectations in non-problem gamblers. A possible interpretation of the diminished responsiveness to low-risk wagers in the problem gamblers, may be that this is due to a diminished reward sensitivity due to a blunted brain response to low-risk monetary rewards.

When summarizing the neuroimaging studies on cue-reactivity in PG and PrG, a convergent picture emerges regarding the studies that employ gambling pictures or gambling movies—in which actual gambling scenes are included. In these studies, increased responsiveness in fronto-striatal reward circuitry and brain areas related to attentional processing towards gambling stimuli is present in pathological gamblers/problem gamblers compared to HCs (Crockford et al., 2005; Goudriaan et al., 2010; Miedl et al., 2010; Wölfling et al., 2011). In contrast, in the one study employing stress-provoking situations, followed by verbal descriptions of wanting to engage in gambling, diminished responsiveness in fronto-striatal circuitry was found (Potenza et al., 2003b). These findings imply that cue-reactivity elicited by gambling stimuli engages reward- and motivation related circuitry thus potentially enhancing the chance of engaging in gambling. On the other hand, negative mood states induced by stressful situations may induce a relatively diminished activity in the same reward- and motivation related circuitry in pathological gamblers, which in turn may elicit craving for gambling, in order to relieve this depletion in reward experience (or anhedonia). The one finding of diminished fronto-striatal reactivity (Potenza et al., 2003b) relates to the “allostatic” negative emotional state (e.g., dysphoria, anxiety, irritability) reflecting a motivational withdrawal syndrome state as hypothesized by Koob and Le Moal and as recently integrated in a review by Koob and Volkow (2010). The remainder of the neuroimging findings in response to gambling cues relate to the preoccupation and anticipation of engaging in addictive behavior, characterized by craving. Thus, both increased responsivity in the brain’s reward system to gambling cues as well as decreased responsivity of the reward system to stress-provoking cues in anticipation of gambling could lead to craving and (relapse in) gambling. This combination is also consistent with a behavioral study by Kushner et al. (2007), in which diminished cue reactivity was reported after negative mood induction.

Together, these cue-reactivity studies and addiction theories indicate that an important area to investigate in PG and PrG is the link between positive mood states and negative mood states/ stress reactivity, and both craving for gambling and gambling behavior. From the studies comparing gambling stimuli to neutral stimuli, increased frontal-striatal reactivity relating to increased cue-reactivity is evident. However, the role of the amygdala and negative emotional mood states (i.e., as a “motivational withdrawal syndrome”) in inducing craving and relapse in PG and PrG should receive additional research attention.

The “withdrawal/negative affect” part of the addiction cycle, which consists of re-engagement in addictive behaviors due to withdrawal effects or negative affect, in order to diminish withdrawal and/or negative affect (Koob and Volkow, 2010) can be linked to the emotionally vulnerable problem gambler, one of the three subtypes of problem gamblers, as proposed by Blaszczynski and Nower (2002) and characterized by stress reactivity and negative mood as a pathway to PrG (Blaszczynski and Nower, 2002). The “preoccupation/anticipation” part of the addiction cycle, which is characterized by enhanced attention and cue-reacitivity towards addiction-relevant cues, links to the “antisocial, impulsivist” subgroup of problem gamblers as defined by Blaszczynski and Nower (2002). They describe the latter subgroup of problem gamblers as characterized by higher impulsivity, and clinical impulsive behaviors such as ADHD and substance abuse, which promote and fasten processes of classical and operant conditioning in developing PrG (Blaszczynski and Nower, 2002). So far, these three subtypes of pathological gamblers have hardly been studied empirically: Ledgerwood and Petry investigated these three gambling subtypes within a group of 229 pathological gamblers, which were based on self-report questionnaires. Although the subtypes differed on PrG severity, subtyping did not predict a differential treatment response. Several behavioral studies indicate differences between problem gamblers and HCs in stress reactivity. For instance, in a recent study (Steinberg et al., 2011), uncontrollable noise (stress induction) led to diminished craving for gambling in problem gamblers, whereas it increased craving for alcohol use in problem gamblers, alcohol use disordered participants and HCs. This finding, although in a small sample (12 participants in each clinical group), indicates that differential changes in craving for different addictive behaviors may result from stress (here: gambling vs. alcohol use). In a self-report study (Elman et al., 2010) the only measure positively related to gambling urges in problem gamblers was a daily stress inventory, indicating a positive relation between stress and craving for gambling. Interestingly, in a recent pilot-study with a pharmacological challenge with yohimbine, significant left amygdala activation in response to yohimbine across all four PG subjects was observed, whereas this effect was not present in the five HCs, suggesting pharmacologically induced stress sensitization in the brain of pathological gamblers. Thus, studies focusing on the relation between stress reactivity and gambling cues, gambling urges, and gambling behavior are needed, in order to elucidate the etiology of both the withdrawal/negative affect (stress reactivity) and the motivation/anticipation (cue reactivity) part of the addiction cycle in PG and PrG. Based on the results of these behavioral and physiological studies, and the negative finding from the one study focusing on the three subtypes of pathological gamblers (Ledgerwood and Petry, 2010), it is clear that more (neuro)biological research is needed into subtyping of PG. It may well be that one problem gambler subtype is identified for whom gambling urges emerge through negative affect (with amygdala circuit abnormalities as a neural mechanism) and another problem gambler subtype where gambling urges emerge through gambling cues (with a hyperactive orbitofronto-striatal circuitry as underlying neural mechanism). This subtyping of pathological gamblers based on endophenotype (negative affect/stress reactivity vs. positive affect/gambling cue reactivity) could then be compared to the three subtypes as defined by Nower and Blaszczynski (2010): behaviorally conditioned, emotionally vulnerable and antisocial-impulsive.

Although a minimal number of neuroscience studies on stress reactivity in PG and PrG exist, a related issue is the presence of either increased or decreased reward sensitivity in neuroimaging studies in PG and PrG, and these studies will be discussed next.

## Excessive or diminished reward sensitivity in problem gambling: is it all in the game or all in the money?

A popular hypothesis of addiction is that substance dependent persons suffer from a reward deficiency syndrome, which makes them pursue strong reinforcers (i.e., drugs) to overcome this deficiency (Comings and Blum, 2000). The first fMRI studies in PG focusing on reward processing have reported results consistent with such decreased reward sensitivity. For example, in response to monetary gains compared to monetary losses pathological gamblers showed blunted activation of the ventral striatum and ventral prefrontal cortex (Reuter et al., 2005). Similarly attenuated activation of ventral prefrontal cortices was present in with a cognitive switching paradigm where problem gamblers could win or lose money dependent on their performance (de Ruiter et al., 2009).

Recently, more detailed studies investigating *different phases of reward processing* have been conducted. Using a modified monetary incentive delay (MID) task (Knutson et al., 2000) in which subjects have to make speeded responses to acquire points/money or to prevent losing points/money, pathological gamblers showed attenuated ventral striatal responses during reward anticipation as well as in response to monetary wins (Balodis et al., 2012; Choi et al., 2012). Whereas results from these two studies are consistent with the reward deficiency hypothesis, other fMRI studies have found increased responses in anticipation of reward or after receiving rewards in fronto-striatal reward related brain areas.

For instance, using a probabilistic choice game to model anticipatory processing, pathological gamblers showed greater dorsal striatum activity during anticipation of large rewards compared to small rewards (van Holst et al., 2012c). In addition, pathological gamblers compared to controls showed higher activity in the dorsal striatum and OFC for gain-related expected value. Hyper-reactivity after receiving monetary rewards in high risk bets was also found in the medial frontal cortex with an ERP study using a black jack task (Hewig et al., 2010). In a fMRI study by Miedl et al. (2012) subjective value coding for delay discounting and probability discounting in pathological gamblers and HCs was investigated. The subjective value for each task was computed for each participant individually and correlated with brain activity in the ventral striatum. Compared to controls, pathological gamblers showed a greater subjective value representation in the ventral striatum on a delay discounting task, but a reduced subjective value representation during the probabilistic discounting task. This indicates that pathological gamblers evaluate values and probabilities differently than controls. These results suggest that abnormal choice behavior with regard to future delayed rewards in problem gamblers could be related to different value coding.

At this point it is unresolved whether PG is associated with hyper- or hypo-activity in the reward circuitry in response to monetary cues, a similar issue that consists in the substance dependence literature (Hommer et al., 2011). Several methodological issues could explain the hyper- or hypo-activity findings in the reward circuitry found in the above mentioned studies. For example, in the MID task subjects have to respond as quickly as possible to a target to obtain a reward whereas in the task used by van Holst et al. (2012c) subjects have no influence on their wins or losses. This difference in control over the task outcomes could have influenced the striatal responses during the task. Furthermore, the graphic designs of the two studies also differed markedly; the MID task used in the study by Balodis et al. (2012) used non-monetary abstract pictograms, the task by van Holst et al. (2012c) featured familiar playing cards and Euro coins and bills. These gambling associated cues may elicit cue reactivity responses leading to hyperresponsivity in the striatal regions (see for a discussion: Leyton and Vezina, 2012; van Holst et al., 2012c, d). This hypothesis regarding diminished reactivity of the striatum in the absence of addiction relevant cues, and an overactivity of the striatum in the presence of addiction relevant cues was recently reviewed in depth by Leyton and Vezina (2013).

The reward deficiency hypothesis of addiction has received considerable support from PET studies measuring dopamine functioning, consistently showing lower dopamine D2/D3 receptor binding potential in drug dependent subjects (Martinez et al., 2004, 2005, 2011; Volkow et al., 2004, 2008; Lee et al., 2009). Whether this D2/D3 receptor binding potential underlies PG is still unclear because PET techniques have only recently been utilized in PG. Currently, no significant differences in baseline DA binding in pathological gamblers compared to HCs seems to be present (Linnet et al., 2010; Joutsa et al., 2012; Boileau et al., 2013) but other studies indicate a positive correlations between DA binding and gambling severity and impulsivity (Clark et al., 2012; Boileau et al., 2013). In addition, A PET study measuring DA activity during the Iowa gambling task found that DA release in pathological gamblers was related to excitement (Linnet et al., 2011a) and poor performance (Linnet et al., 2011b). Overall these results do suggest a role for abnormal DA binding in PG but not to the same extent as that found in drug addiction in which clear diminished binding potentials are consistently reported (Clark and Limbrick-Oldfield, 2013). Missing from the literature are studies measuring more stable baseline DA synthesis capacity: existing studies have only focused on aspects related to highly state dependent DA D 2/3 receptor availability. Studies measuring DA synthesis capacity could test the hypothesis of a higher DA synthesis capacity in PG and PrG. Higher DA synthesis could lead to higher dopaminergic *reactivity* when confronted with addiction related cues (e.g., games, money, risk). Furthermore, PG studies directly manipulating DA and measuring fMRI BOLD responses during reward processing could provide important information about the causal role of DA in PG.

An alternative hypothesis, next to the reward deficiency hypothesis for PG and PrG is that, similar to substance use disorders (SUDs; Robinson and Berridge, 2001, 2008), pathological gamblers and problem gamblers suffer from an enhanced incentive salience for gambling related cues. This enhanced incentive salience for gambling cues could be so strong that it overrides incentive salience of alternative sources of reward, leading to an imbalance in incentive motivation. To test whether pathological gamblers would suffer from an overall reward deficiency or from an imbalance in incentive salience, Sescousse et al. (2013) compared neural responses to both financial gains and to primary rewards (erotic pictures) in pathological gamblers and HCs. In line with the latter hypothesis, hypo-reactivity was observed for the erotic cues, in contrast with normal-reactivity to the financial rewards, indicating an imbalanced incentive salience attribution in PG. Taken all the above studies together, at this point it seems most likely that pathological gamblers are not suffering from a reward deficiency in general but that pathological gamblers have a different appraisal of gambling related stimuli, presumably caused by enhanced incentive salience of gambling related stimuli.

Recently fMRI studies have focused on specific gambling related cognitive biases. This is important because problem gamblers often display a number of cognitive biases regarding gambling games (Toneatto et al., 1997; Toneatto, 1999; Clark, 2010; Goodie and Fortune, 2013). For example, gamblers are known to falsely believe that they can influence outcome probabilities of games (“illusion of control”) (Langer, 1975). Various intrinsic features of games of chance promote these biases (Griffiths, 1993), as for example “near-miss” events (Kassinove and Schare, 2001). These near-wins or near-miss outcomes (which are actually losses) occur when two reels of a slot machine display the same symbol and the third wheel displays that symbol immediate above or below the pay-off line. A study investigating near-miss effects in problem gamblers found that brain responses during near-miss outcomes (compared to full-miss outcomes) activated similar brain reward regions such as the striatum and insular cortex as during win outcomes (Chase and Clark, 2010). Habib and Dixon (2010) found that near-miss outcomes lead to more win-like brain responses in pathological gamblers, whereas HCs activated brain regions associated with losses to a larger extent. These studies contribute to a better understanding of the addictiveness of gambling games and its underlying neuronal mechanism.

## Can enhanced salience for gambling related stimuli lead to loss of control over behavior?

An influential and empirically grounded neurobiological model for substance dependence, the Impaired Response Inhibition and Salience Attribution (I-RISA) model, postulates that repeated drug use triggers a series of adaptations in neuronal circuits involved in memory, motivation, and cognitive control (Volkow et al., 2003). If an individual has used drugs, memories of these events are stored as associations between the stimulus and the elicited positive (pleasant) or negative (aversive) experiences, facilitated by dopaminergic activation caused by the drug of abuse. This results in an enhanced (and long-lasting) salience for the drug and its associated cues at the expense of decreased salience for natural reinforcers (Volkow et al., 2003). In addition, the I-RISA model assumes loss of control (disinhibition) over drugs due to enhanced salience and pre-existing deficiencies (as discussed in part 1 of the review), which renders individuals suffering from addictive disorders vulnerable to relapse into addictive behavior.

In addictive disorders including PG, there is evidence that both affective and motivational systems are more sensitive to addiction relevant material. For example, studies have shown that addiction related cues attract more attention than other salient stimuli, a phenomenon known as “attentional bias” (McCusker and Gettings, 1997; Boyer and Dickerson, 2003; Field and Cox, 2008). As discussed in the “cue reactivity” section of this review, in problem gamblers, enhanced brain responsiveness towards gambling related cues (“cue reactivity”) has also been found in brain areas related to motivational processing and cognitive control (amygdala, basal ganglia, ventrolateral prefrontal cortex and dorsolateral prefrontal cortex; Crockford et al., 2005; Goudriaan et al., 2010).

As discussed in the first section of this review, PG is associated with impaired cognitive control. However how cognitive control interacts with motivational processes is still subject of investigation. Just recently, studies have started to test the interaction between cognitive control and salience attribution in PG. In one of our recent studies, we employed a modified Go/NoGo task by including affective stimulus blocks (gambling, positive and negative), in addition to the standard affectively neutral block in problem gamblers and HCs (van Holst et al., 2012b). Subjects were requested to respond or withhold a response to specific types of pictures with a different emotional loading, allowing the investigation of the interaction between motor inhibition and salience attribution. Whereas we found no behavioral differences on neutral response inhibition trials, problem gamblers compared to controls showed greater dorsolateral prefrontal and ACC activity. In contrast, during gamble and positive pictures problem gamblers made less response inhibition errors than controls and showed reduced activation of the dorsolateral prefrontal and ACC. This study indicated that pathological gamblers rely on compensatory brain activity to achieve similar performance during neutral response inhibition. However, in a gambling-related or positive context response inhibition appears to be *facilitated*, as indicated by lower brain activity and fewer response inhibition errors in pathological gamblers. Data from this Go/NoGo study was further analyzed to test the effect of affective stimuli on functional connectivity patterns during the task (van Holst et al., 2012a). As expected, adequate response inhibition was related to functional connectivity within the sub-regions of the dorsal executive system as well as on functional connectivity between the dorsal executive and the ventral affective system in both HCs and problem gamblers. Compared to HCs, problem gamblers showed a stronger positive correlation between the dorsal executive system and task accuracy during inhibition in the gambling condition. These findings suggest that increased accuracy in pathological gamblers during the gambling condition was associated with increased connectivity with the dorsal executive system (van Holst et al., 2012a). It seems likely that DA function plays an important role in these findings. Salient stimuli enhance DA transmission in the mesolimbic system (Siessmeier et al., 2006; Kienast et al., 2008) and DA is known to modulate prefrontal cortex functioning (Robbins and Arnsten, 2009). Indeed, in humans, DA transmission has an effect on functional connectivity within the corticostriatal thalamic loops (Honey et al., 2003; Cole et al., 2013). More research is needed to further clarify the interaction between motivation, DA and cognitive control in PG. In the earlier mentioned review by Leyton and Vezina (2013), a model is proposed that integrates the influence of these opposite striatal responses on the expression of addictive behaviors. Central to his model is the idea that low striatal activity leads to an inability to sustain focussed goal-directed behavior, whereas in the presence of high striatal activity (when drug cues are present) a sustained focus and drive to obtain rewards is present. The findings reviewed above (van Holst et al., 2012a, b) fit this model well: better performance was present in problem gamblers in the positive and gambling conditions, and more functional connectivity was found with the dorsal executive system in problem gamblers in the gambling condition. This could be an indication of normalization in probleml gamblers of the underactive striatal system, in the presence of salient motivational cues in the positive and gambling Go/NoGo conditions.

It is clinically relevant to further investigate whether increased activity in the reward system indeed has the effect of transiently restoring prefrontal cortex functioning in problem gamblers. This could be tested by pharmacological challenges or by enhancing activity in the reward system more locally, for example by using real time-fMRI neurofeedback (deCharms, 2008) or Transcranial Magnetic Stimulation (TMS; Feil and Zangen, 2010). However, we suggest that enhanced salience to rewarding stimuli could also lead to *impaired* task performance. For example, when too much attention is allocated to salient stimuli, this can result in attenuated executive control recourses (Pessoa, 2008). Enhanced reward seeking behavior and enhanced responsiveness to potential rewards could therefore be an important concept in understanding why especially on tasks with contingencies gamblers show diminished cognitive performance (Brand et al., 2005; Goudriaan et al., 2005, 2006; Labudda et al., 2007; Tanabe et al., 2007; de Ruiter et al., 2009).

## Summary neuroimaging findings: self-control, cue-reactivity, reward sensitivity at different stages of gambling, and the interaction between self-control and motivational urge

When trying to reach an overarching conclusion with regard to the studies reviewed, it is clear that for some topics, consistent findings have been established over the years. For instance, the notion of increased impulsivity in PG and PrG is firmly established and the first neuroimaging studies show that this heightened impulsivity is accompanied by diminished prefrontal and ACC functioning. It is clear that the field of cognitive functions in PG needs more neuroimaging studies to investigate what cognitive functions are most affected. Neuroimaging cue-reactivity studies indicate that when gambling cues are present, the motivational system of the brain is overactive in PG and PrG, as evidenced in higher parahippocampal, amygdala, basal ganglia, and OFC activation. With regard to either enhanced neural reward sensitivity or diminished reward sensitivity, the first studies seem to indicate that whereas enhanced activation of the brain’s reward circuitry is present in *anticipation of* winning or in experiencing risky gamble situations, diminished reward responsiveness is present in this same circuitry *after* winning and/or losing money. Finally, the interaction of cue-reactivity and cognitive control suggests that the activation of the cognitive control system in problem gamblers may be enhanced by activating the motivational circuit. However, this finding is in need of replication, and the role of DA in facilitating or diminishing cognitive control in PG deserves further study.

## Clinical implications

Cognitive behavioral therapy (CBT) for problem gamblers focuses on behavioral and cognitive interventions to curb the motivational lure of gambling behavior and has been shown to be effective in the treatment of PG (Petry, 2006; Petry et al., 2006), although relapse is still high, ranging around 50–60% in treatment studies, with rates of continuous abstinence for a year as low as 6% (Hodgins et al., 2005; Hodgins and el Guebaly, 2010). Thus, there is still room for major improvement in treatment results for PG/PrG. CBT focuses on enhancement of cognitive control over gambling, and a change in the behavior of engagement in gambling due to encountering gambling cues or experiencing craving. Specific techniques used in CBT for PG and PrG include learning coping strategies, applying stimulus control strategies, and handling high risk situations by implementing behavioral strategies, for instance on emergency cards. Thus, in CBT for PG and PrG, a substantial part of the intervention depends on engagement of executive functions by implementing behavior and emotion regulation strategies. In other psychiatric disorders, neuroimaging studies have shown that differences in pre-treatment brain functioning can predict CBT treatment effects. For instance, better frontal-striatal brain functions during a response inhibition task resulted in better response to CBT in post traumatic stress disorder (Falconer et al., 2013). Increased activity at baseline in the ventromedial PFC as well as valence effects in emotional tasks (e.g., social threat tasks) in the (anterior) temporal lobe, ACC and DLPFC promote treatment success in major depressive disorder (Ritchey et al., 2011) and in social anxiety disorder (Klumpp et al., 2013). These findings not only suggest that brain functions may be important new biomarkers for indicating the chance for treatment success with CBT, but also point to the potential value of new interventions targeting neurobiological vulnerabilities of PG and PrG. By studying brain functions that are biomarkers for CBT success in PG and subsequently improving these brain functions by neuromodulation or pharmacological interventions, treatment results for PG and PrG may improve.

Several interventions targeted at neurobiological vulnerabilities of PG and PrG are promising and may result in additional treatment effects by interacting and improving the functions that are a prerequisite for CBT success. Recently, neuromodulation interventions have gained interest in addiction research. Specifically, neurostimulation methods such as repeated Transcranial Magnetic Stimulation (rTMS) and transcranial Direct Current Stimulation (tDCS) were evaluated in a meta-analysis (Jansen et al., 2013). From this meta-analysis, a medium-effect size was found for neurostimulation with either rTMS or tDCS to reduce craving for substances or high-palatable food. In a study with multiple sessions of rTMS in 48 heavy smokers, 10 daily sessions of active rTMS over the DLPFC resulted in diminished cigarette consumption and nicotine dependence, compared to a control condition of sham rTMS (Amiaz et al., 2009). Related to neurostimulation, EEG neurofeedback in SUDs has recently gained renewed interest, with some pilot studies showing positive results of EEG neurofeedback training in cocaine dependence (Horrell et al., 2010) and opiate dependence (Dehghani-Arani et al., 2013). Thus, interventions with neurostimulation or neurofeedback in PG and PrG are warranted as well, to investigate whether neurostimulation interventions also hold promise in this behavioral addiction.

As a potential non-pharmacological intervention, changes in the motivational system in PG could be targeted by “attentional retraining” (MacLeod et al., 2002; Wiers et al., 2006). During attentional retraining patients are trained to reverse their attentional bias by performing computer tasks, thus aiming to reduce cue reactivity and to change habitual behaviors. A related intervention is retraining of automatic action tendencies, in which approach behavior towards addiction related stimuli is retrained to avoidance behavior (Wiers et al., 2006, 2010; Schoenmakers et al., 2007). In alcohol use disorders, results from the suggested interventions are promising (Wiers et al., 2006, 2010). However, these interventions have not yet been tested in PG and long-term effects of attentional and action tendency retraining are not yet available and need to be assessed in future research.

## Pharmacological interventions

In addition to the potential of neurostimulation, neurofeedback and attentional retraining interventions, a number of promising pharmacological interventions for the treatment of PG have been reported (for a review see van den Brink, 2012). Neurobiological findings indicate a pivotal role of the mesolimbic pathway, comprising the ventral striatum, and ventromedial prefrontal cortex (VMPFC) in PG. Because the VMPFC is a structure that mainly depends on DA projections that communicate with limbic structures to integrate information, dysfunctional DA transmission could be the underlying deficit causing the VMPFC dysfunctions in PG. However, numerous other neurotransmitter systems are probably also engaged and may interact during the processing of positive and negative feedback. For example, opiates are known to increase DA release in the reward pathway, and the opiate antagonists naltrexone and nalmefene, which are known to decrease DA release, have been found to reduce reward sensitivity and probably increase punishment sensitivity as well (Petrovic et al., 2008). Moreover, treatment with opiate antagonists has been shown to be effective in PG and to diminish gambling urges (Kim and Grant, 2001; Kim et al., 2001; Modesto-Lowe and Van Kirk, 2002; Grant et al., 2008a, b, 2010b).

Whereas in substance addictions, drugs and drug-associated stimuli may elicit DA release in the ventral striatum and reinforce drug intake during the acquisition of a substance use disorder, *chronic* drug intake is associated with neuroadaptation of glutamatergic neurotransmission in the ventral and dorsal striatum and limbic cortex (McFarland et al., 2003). In addition, cue exposure has been found to depend on projections of glutamatergic neurons from the prefrontal cortex to the nucleus accumbens (LaLumiere and Kalivas, 2008). Blocking the release of glutamate has prevented drug seeking behavior in animals as well as in human substance dependent persons (Krupitsky et al., 2007; Mann et al., 2008; Rösner et al., 2008). Therefore, the first promising results from pilot studies with N-acetyl cysteine (Grant et al., 2007) and memantine (Grant et al., 2010a), which modulate the glutamate system, warrant larger studies that investigate the effects of these glutamate regulating compounds in the treatment of PG.

Besides the focus on improving cognitive functions and diminishing craving by neuromodulation or pharmacological techniques, recently, interest in the influence of protective factors has grown. For instance, low impulsivity and active coping skills have been linked to a more positive outcome for SUDs. Thus, not only a focus on risk factors, but also on the role of protective factors and environmental variables that promote them may foster our understanding of the brain-behavior relationships and the pathways in developing and recovering from PG and PrG. A potential application of a focus on both risk and protective factors may be to monitor cognitive-motivational and brain functions during treatment, investigate which functions spontaneously normalize, and which functions need additions from novel interventions such as cognitive training, neuromodulation, or pharmacological interventions.

## Conclusions

PG and PrG are clearly associated with cognitive and motivational differences in neuropsychological and brain functioning. Specifically, higher impulsivity and impaired executive functioning is present, which is associated with diminished functioning of the cognitive control circuitry in the brain, such as the ACC and dorsolateral prefrontal cortex. In addition, motivational functions are affected, which are associated with differential functioning in medial frontal areas and in the thalamo-striatal circuitry, linking to the frontal cortex. More research is needed to investigate the interaction between cognitive and motivational functions, as the combination of gambling cues in cognitive tasks sometimes also improves cognitive functions. Investigating the efficacy of novel interventions that target these neurobiological mechanisms, such as neuromodulation, cognitive training, and pharmacological interventions, is needed in order to investigate its potential to improve treatment outcome. In addition, research focusing on protective factors and the spontaneous recovery of risk factors could indicate which mechanisms to target in order to improve the course of PG.

## Author contributions

Anna E. Goudriaan, Murat Yücel, and Ruth J. van Holst contributed to the design of the review, Anna E. Goudriaan and Ruth J. van Holst drafted parts of the manuscript, Anna E. Goudriaan, Ruth J. van Holst, and Murat Yücel revised this work critically for important intellectual content. Final approval of the version to be published was given by all authors and all authors agree to be accountable for all aspects of the work in ensuring that questions related to the accuracy or integrity of any part of the work are appropriately investigated and resolved.

## Conflict of interest statement

The authors declare that the research was conducted in the absence of any commercial or financial relationships that could be construed as a potential conflict of interest.
